# Response to ‘*Helicobacter pylori* infection of AZ-521 cells reveals a type IV secretion defect and VacA-independent CagA phosphorylation’

**DOI:** 10.1242/dmm.032821

**Published:** 2017-12-01

**Authors:** Masayuki Nakano, Toshiya Hirayama

**Affiliations:** 1 Department of Bacteriology, Institute of Tropical Medicine, Nagasaki University, Nagasaki 852-8523, Japan; 2 Department of International Health, Institute of Tropical Medicine, Nagasaki University, Nagasaki 852-8523, Japan

We are writing in response to the Correspondence from Tegtmeyer and Backert regarding the article by Nakano et al. (2016).

Two virulence factors, cytotoxin-associated gene A (CagA) and vacuolating cytotoxin (VacA), are required for pathological actions in *Helicobacter pylori*-infected cells (Isomoto et al., 2010; Hatakeyama, 2014). After delivery of CagA into host cells by a type IV secretion system (T4SS), it is tyrosine phosphorylated at Glu-Pro-Ile-Tyr-Ala (EPIYA) motifs by Src and Abl family kinases. Phosphorylated CagA is responsible for subsequent biological actions in infected cells, such as modulation of signal transduction pathways (Odenbreit et al., 2000; Mueller et al., 2012). Previous studies have defined the molecular mechanism of CagA phosphorylation in host cells. CagL, a component of T4SS, a key participant in CagA phosphorylation pathways, binds to host cell protein β1 integrin, suggesting that this interaction is involved in the pathological actions of CagA in host cells (Kwok et al., 2007). Receptor protein tyrosine phosphatase alpha (RPTPα), a VacA receptor, has been shown to contribute to activation of c-Src and Src family kinases and serves as a physiological regulator of Src family kinases (Su et al., 1999).

**Fig. 1.**
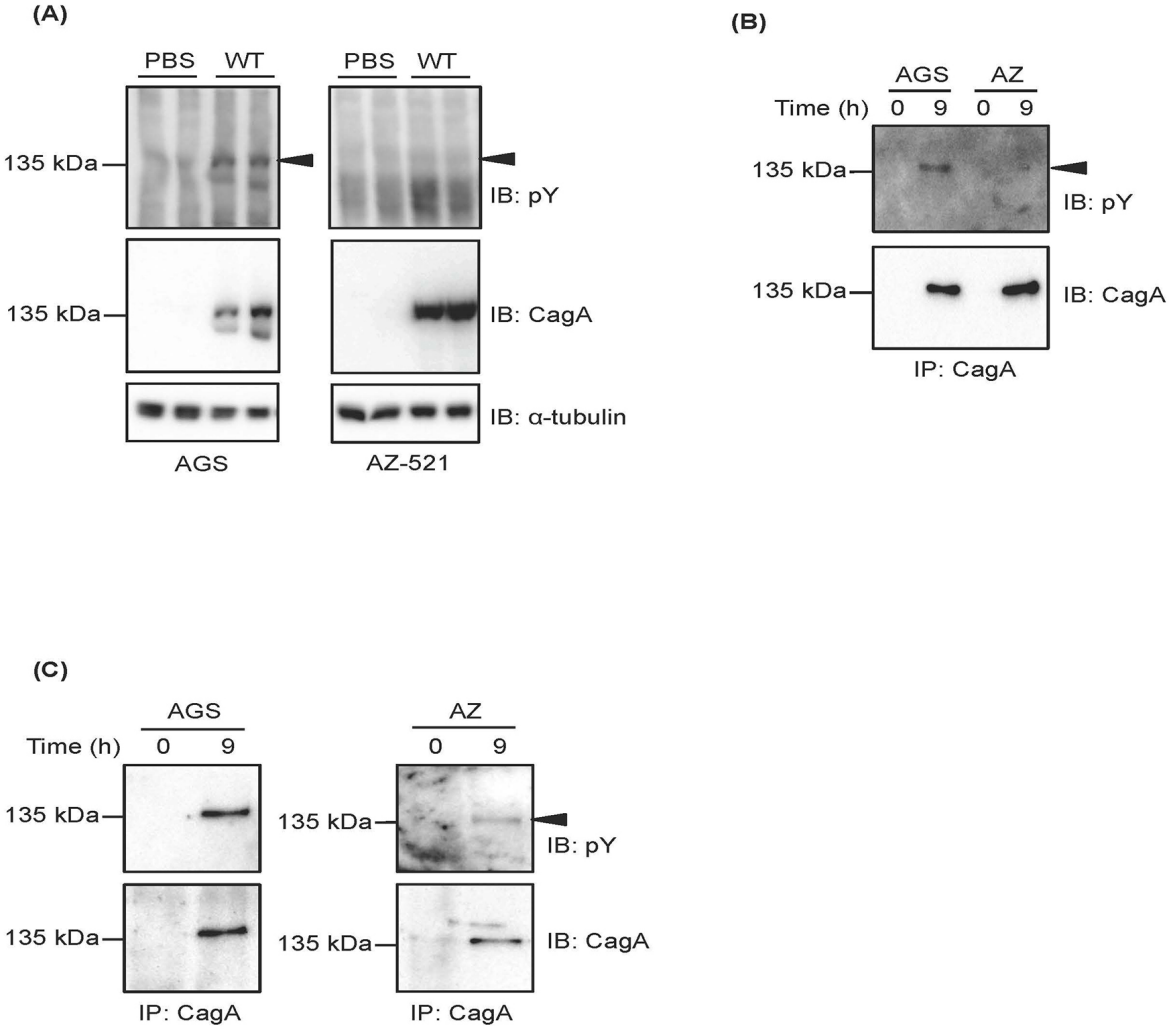
**Detection of total CagA and pCagA proteins in AGS and AZ-521 cells.** (A) After infection of AGS and AZ-521 cells with *H. pylori* strain ATCC43504 (WT) at a multiplicity of infection of 100, cell lysates were prepared and loaded on acrylamide gel. Following transfer of proteins to the membrane, signals were detected by standard western blotting. (B,C) After infection of AGS and AZ-521 cells with a WT strain and preparation of cell lysates, immunoprecipitations using anti-CagA antibody were performed to concentrate translocated CagA proteins in host cells as previously described (Nakano et al., 2016). Then, pCagA was detected on the same membrane (B) or on separate membranes (C). To detect the signal in both cell lines, we used 200 µg (AGS) and 500 µg (AZ-521) of whole-cell lysates for immunoprecipitation (C). Signals were detected using anti-phosphotyrosine (pY) and anti-CagA antibodies; α-tubulin served as a loading control. Arrowheads represent the signal of phosphorylated CagA. IB, immunoblotting; IP, immunoprecipitation; PBS, phosphate buffered saline.

Tegtmeyer and Backert showed in their Fig. 1A that phosphorylated CagA (pCagA) was observed in AGS cells during infection with *H. pylori*, but not in AZ-521 cells. We agree with their observations; we also observed that pCagA could not be detected in AZ-521 cells by standard western blotting using anti-pY antibody during infection with *H. pylori* (Fig. 1A). To address this observation, we performed immunoprecipitation using anti-CagA antibody to concentrate translocated pCagA proteins in AZ-521 cells. We wanted to know whether pCagA during *H. pylori* infection could be detected in AZ-521 cells. When we carried out the infection assay in AZ-521 cells by challenging with *H. pylori* strains under several experimental conditions, we could detect pCagA by infection with wild-type *H. pylori*. On the other hand, this signal could not be detected during infection with a *cag*PAI-deleted mutant of *H. pylori*, indicating that this signal originated with pCagA or total CagA protein (fig. 4A in Nakano et al., 2016). In addition, the presence of pCagA in AZ-521 cells during infection with *H. pylori* was verified by two additional methods (fig. 4A and B in Nakano et al., 2016). First, we examined the amount of SHP2 phosphatase by immunoprecipitation using anti-CagA antibody. Previous studies have shown that SHP2 phosphatase specifically binds to pCagA in *H. pylori*-infected host cells, suggesting that the amount of precipitated SHP2 phosphatase correlated with the amount of pCagA (Higashi et al., 2002). Second, we verified our results by examining the amount of pCagA using an antibody that recognizes phosphorylated Tyr972 in CagA, a major c-Src phosphorylation site (Backert et al., 2001; Kwok et al., 2007). Thus, these two findings support our result on the amount of pCagA. As shown in Fig. 1C, we found that Tegtmeyer and Backert also detected a corresponding pCagA signal in AZ-521 cells. Although total translocated CagA protein, precipitated with anti-CagA antibody, was similar in both AGS and AZ-521 cells, the amount of pCagA observed in AGS cells was larger than that seen in AZ-521 cells. We also showed this larger difference between AGS and AZ-521 cells, related to the phosphorylation level of translocated CagA, by immunoprecipitation using anti-CagA antibody (Fig. 1B). When we detected pCagA after immunoprecipitation on the separate membranes, pCagA in AGS cells could be detected in a very short period of time. But, detection of pCagA in AZ-521 cells required a longer period of time and a larger amount of protein than that required in AGS cells (Fig. 1C). Therefore, we think that it is difficult to evaluate the level of signal intensities of pCagA from AZ-521 cells when cell lysates derived from AGS and AZ-521 cells are loaded on the same membrane. As shown in Fig. 1D by authors of the Correspondence, signals of pCagA from AGS cells are much stronger than those seen in AZ-521 cells and, therefore, stronger signals obtained from AGS cells have an impact on the evaluation of signal intensities of pCagA obtained from AZ-521 cells. It is best to use separate gels with AGS cells and AZ-521 cells to obtain more easily validated signals.

The physiological concentration of VacA during *H. pylori* infection is not known. Therefore, it is not clear whether the concentrations of VacA used in our study correspond to the relative concentration on the surface of *H. pylori*-infected host cells. Although VacA concentrations that affect its biological functions in host cells, and physiological concentration during *H. pylori* infection, should be examined in further studies, we propose that VacA does not have a prolonged effect on induction of Src phosphorylation in AZ-521 cells (fig. 1A in Nakano et al., 2016). We observed Src phosphorylation induced by VacA after 1 h incubation with purified VacA, and incubation of AZ-521 cells with VacA for 2 h resulted in diminished signal of Src phosphorylation under some experimental conditions, indicating that Src phosphorylation induced by VacA in AZ-521 cells might be observed at certain points in time. Detailed molecular mechanisms of Src phosphorylation induced by VacA should be evaluated in further studies.

We also think that AGS cells represent a good model gastric epithelial cell line to study the biological functions of CagA in *H. pylori*-infected host cells. Although there appears to be a problem regarding CagA phosphorylation, we used AZ-521 cells because this cell line is highly sensitive to VacA-induced actions and expresses several VacA receptors including RPTPα (Isomoto et al., 2010; Radin et al., 2011). But, as Tegtmeyer and Backert mentioned, monolayers of AZ-521 cells and AGS cells might not reflect the precise physiological actions of stomach epithelia. It is difficult to reconstruct detailed environments mimicking host cell surfaces during *H. pylori* infection using these cell lines. Primary cell cultures and polarized cells with 3D structure, such as organoids generated from the biopsy of human gastric epithelium or animal experimental models, might be applicable for studies regarding the biological functions of virulence factors such as CagA and VacA in *H. pylori*-infected host cells.
